# Supplementary material for: A Critical View of the Application of the APEX Software (Aqueous Photochemistry of Environmentally-Occurring Xenobiotics) to Predict Photoreaction Kinetics in Surface Freshwaters
Source: Molecules. 2019 Dec 18;25(1):9. doi: 10.3390/molecules25010009 (PMC7017383; doi:10.3390/molecules25010009)
Supplement: Supplementary file 1 [file molecules-25-00009-s001.zip › 3.2.4_gcc-4.4.0.zip!/3.2.4_gcc-4.4.0/3.2.4_gcc-4.4.0/doc/octave/html/interpreter/Indexing-Cell-Arrays.html]

Indexing Cell Arrays - Untitled


Next: Cell Arrays of Strings,
Previous: Creating Cell Arrays,
Up: Cell Arrays


---

#### 6.2.3 Indexing Cell Arrays

As shown in see Basic Usage of Cell Arrays elements can be
extracted from cell arrays using the { and }
operators. If you want to extract or access subarrays which are still
cell arrays, you need to use the ( and ) operators. The
following example illustrates the difference:

```
     c = {"1", "2", "3"; "a", "b", "c"; "4", "5", "6"};
     c{2,3}
          => ans = c
     
     c(2,3)
          => ans =
             {
               [1,1] = c
             }
```

So with {} you access elements of a cell
array, while with () you access a sub array of a cell
array.

Using the ( and ) operators, indexing works for cell
arrays like for multidimensional arrays. As an example, all the rows
of the first and third column of a cell array can be set to `0`
with the following command:

```
     c(:, [1, 3]) = {0}
          =>  =
             {
               [1,1] = 0
               [2,1] = 0
               [3,1] = 0
               [1,2] = 2
               [2,2] =  10
               [3,2] =  20
               [1,3] = 0
               [2,3] = 0
               [3,3] = 0
             }
```

Note, that the above can also be achieved like this:

```
     c(:, [1, 3]) = 0;
```

Here, the scalar 0 is automatically promoted to
cell array {0} and then assigned to the subarray of `c`.

To give another example for indexing cell arrays with (), you
can exchange the first and the second row of a cell array as in the
following command:

```
     c = {1, 2, 3; 4, 5, 6};
     c([1, 2], :) = c([2, 1], :)
          => =
             {
               [1,1] =  4
               [2,1] =  1
               [1,2] =  5
               [2,2] =  2
               [1,3] =  6
               [2,3] =  3
             }
```

Accessing multiple elements of a cell array with the { and
} operators will result in a comma-separated list of all the
requested elements (see Comma Separated Lists). Using the
{ and } operators the first two rows in the above
example can be swapped back like this:

```
     [c{[1,2], :}] = deal(c{[2, 1], :})
          => =
             {
               [1,1] =  1
               [2,1] =  4
               [1,2] =  2
               [2,2] =  5
               [1,3] =  3
               [2,3] =  6
             }
```

As for struct arrays and numerical arrays, the empty matrix []
can be used to delete elements from a cell array:

```
     x = {"1", "2"; "3", "4"};
     x(1, :) = []
          => x =
             {
               [1,1] = 3
               [1,2] = 4
             }
```

The following example shows how to just remove the contents of cell
array elements but not delete the space for them:

```
     x = {"1", "2"; "3", "4"};
     x{1, :} = []
     => x =
           {
             [1,1] = [](0x0)
             [2,1] = 3
             [1,2] = [](0x0)
             [2,2] = 4
           }
```
